# Level of perceived social support, and associated factors, in combat-exposed (ex-)military personnel: a systematic review and meta-analysis

**DOI:** 10.1007/s00127-024-02685-3

**Published:** 2024-05-21

**Authors:** Laura E. Grover, Charlotte Williamson, Howard Burdett, Laura Palmer, Nicola T. Fear

**Affiliations:** 1 King’s College London, King’s Centre for Military Health Research, London, SE5 9RJ UK; 2 Academic Department of Military Mental Health, King’s College London, London, SE5 9RJ UK

**Keywords:** Perceived social support, Combat, Military, Post-deployment, Systematic review, Meta-analysis

## Abstract

**Purpose:**

Combat deployment increases exposure to potentially traumatic events. Perceived social support (PSS) may promote health and recovery from combat trauma. This systematic review and meta-analysis aimed to synthesize studies investigating the level of PSS and associated factors among (ex-)military personnel who served in the Iraq/Afghanistan conflicts.

**Methods:**

Five electronic databases were searched in August 2023 and searches were restricted to the beginning of the Iraq/Afghanistan conflicts in 2001. The search was conducted according to the Preferred Reporting Items for Systematic Reviews and Meta-Analyses (PRISMA) guidelines. A quality assessment was carried out, and a meta-analysis and narrative synthesis were performed.

**Results:**

In total, 35 papers consisting of 19,073 participants were included. Of these, 31 studies were conducted in the United States (US) and 23 were cross-sectional. The pooled mean PSS score was 54.40 (95% CI: 51.78 to 57.01). Samples with probable post-traumatic stress disorder had a lower mean PSS score (44.40, 95% CI: 39.10 to 49.70). Approximately half of the included studies (n = 19) investigated mental health in relation to PSS, whilst only four explored physical health. The most frequently reported risk factors for low PSS included post-traumatic stress disorder, depression and anxiety, whilst post-traumatic growth and unit support were protective factors.

**Conclusion:**

Higher levels of PSS were generally associated with more positive psychosocial and mental health-related outcomes following deployment. PSS should be targeted in psychosocial interventions and education programmes. Future research should investigate PSS in (ex-)military personnel across other countries and cultures, based on the lack of studies that focused on PSS in countries outside of the US.

**Supplementary Information:**

The online version contains supplementary material available at 10.1007/s00127-024-02685-3.

## Introduction

Combat operations during the Iraq/Afghanistan conflicts involved participation in, and witnessing of, life-threatening incidents [1]. For some military personnel, this included contact with improvised explosive devices and exposure to ambushes [2]. Combat deployment increases exposure to potentially traumatic events. This can lead to increased risk for combat-related health consequences among (ex-)military personnel.^1^ Health consequences can include post-traumatic stress disorder (PTSD) [3], traumatic brain injury (TBI) [4] or major limb trauma [5]. One potential strategy to reduce health and wellbeing complications following combat exposure may be to increase the availability of social support [6].

Social support is defined within the psychological and epidemiological literature as the availability and adequacy of social connections, comprising structural (received) and functional (perceived) support [7]. Structural social support refers to the presence of relationships, which includes objective measures such as the size of social network, whilst functional social support incorporates quality of support, for example, how successfully a relationship fulfils one’s needs [7, 8]. Sources of support can include family, friends, partners and colleagues. Social support can be further divided into emotional, tangible and informational support [9]. These consist of showing concern for one’s feelings, sharing material or financial resources and providing advice or information, respectively. Perceived social support (PSS) encompasses an individual’s *beliefs* about the scope and suitability of support they experience [10]. Evidence suggests that PSS is more predictive of wellbeing outcomes than structural measures [7, 11, 12]. Therefore, the focus of this review will be on PSS.

A growing body of evidence posits that PSS is one of the key determinants of health and wellbeing across a variety of populations and contexts [7, 13], whilst low PSS has been associated with increased morbidity and mortality [14, 15]. Military samples may have unique experiences of PSS compared to the general population as comradeship and collectivism are central to military culture and are essential for operational success [16]. In a sample of United States (US) (ex-)military personnel who experienced major limb trauma during Iraq/Afghanistan deployment, almost one third (27%) reported low levels of PSS [17].

There is a need to focus research efforts on (ex-)military personnel who served in Iraq/Afghanistan, as survival rates were higher than in previous conflicts due to technological and medical advances [18]. This population may therefore be prone to experiencing psychosocial, occupational and health-related difficulties during deployment and thereafter [19], which may be alleviated by PSS [20]. Studies have investigated PSS during deployment [21], however less is known about PSS following deployment. The post-deployment period may give rise to additional challenges. For example, some personnel were required to leave their military role having sustained a combat-related mental or physical injury [4, 19]. Studies have shown that experiencing medical discharge or involuntary termination is associated with reduced social group engagement [22, 23]. Furthermore, in a sample of (ex-)military personnel who served in Iraq/Afghanistan, loss of brotherhood was reported as the most prevalent challenge to cope with upon returning home [24]. Accordingly, there is a need to understand the social experiences of this population. A thorough investigation may be useful for policy makers and service providers in devising targeted messages and informing psychosocial intervention, to meet the needs of combat-exposed (ex-)military personnel.

To date, no review has investigated PSS in combat-exposed (ex-)military personnel. Therefore, this systematic review and meta-analysis was directed by the following research questions:

1. What levels of PSS are reported in combat-exposed (ex-)military personnel who served in Iraq/Afghanistan?
2. What factors are associated with PSS in combat-exposed (ex-)military personnel who served in Iraq/Afghanistan?

## Methods

This review was prospectively registered with PROSPERO (CRD42023389759) and was conducted according to PRISMA guidelines [25].

### Search strategy

The following five bibliographic databases were searched in January 2023 and re-searched in August 2023: Embase, Medline, PsycINFO, Social Policy and Practice and Applied Social Sciences Index and Abstracts (ASSIA). The search contained key words relating to: “military personnel” and “combat” and “perceived social support” (Appendix 1). Search terms were developed with reference to published literature and following consultation with a librarian. Medical Subject Headings (MeSH) terms were included where available. Searches were restricted from 2001 onwards (beginning of the Iraq/Afghanistan conflicts). Reference lists from eligible studies were searched manually and forward citation tracking was performed.

### Eligibility criteria

#### Inclusion criteria

- Measured PSS in (ex-)military personnel who deployed on a combat operation in Iraq/Afghanistan.
- Peer-reviewed publications, quantitative study design.
- Used a validated measure of PSS ≥ 6 months post-deployment to cover the period after homecoming and beyond [26].
- Published in the English language with full-text availability.

#### Exclusion criteria

- Non-peer reviewed literature, meta-analyses, literature reviews, experimental studies and qualitative studies.
- Measured PSS < 6 months post-deployment.
- Investigated PSS from the perspective of families, carers or healthcare professionals.
- Measured structural social support, for example size of social network, marital status or living arrangements.
- Investigated formal or paid support, for example counselling therapy.

### Screening and data extraction

L.E.G. performed the search, removed duplicates using Zotero reference manager and completed title and abstract screening followed by full-text screening. C.W. independently reviewed 10% of the records during title and abstract (n = 120) and full-text (n = 36) screening stages. A standardised data extraction form was completed independently by L.E.G., and 10% by C.W. (n = 4). Any disagreements were discussed and resolved. The following data were extracted: study information, PSS measure, mean level of PSS and estimates for all investigated factors including size and direction of effect. Effect estimates primarily included odds ratios and regression coefficients. Estimates were extracted for fully adjusted models where available.

### Quality assessment

Methodological quality was assessed using the National Heart, Lung and Blood Institute (NHLBI) quality assessment tool for observational cohort and cross-sectional studies [27]. L.E.G. and C.W. independently performed the quality assessment and discussed any discrepancies. For each included study, 14 questions relating to study quality were answered and articles were defined as “good”, “fair” or “poor” based on the NHLBI quality assessment criteria.

### Data analysis and synthesis

To assess the level of PSS, a meta-analysis was conducted using the statistical software package Stata version 17. A pooled mean PSS score was generated for 13 (out of 15) studies using the Deployment Risk and Resilience Inventory-Post-deployment Social Support-1 (DRRI-PDSS-1) measure [28]. Two studies which used the DRRI-PDSS-1 were excluded from the meta-analysis as the mean level of PSS was not reported [29] and another had substantial missing data [30]. Analysis was not possible with the additional PSS measures due to limited data.

Two subgroup analyses were performed. The first assessed the level of PSS among (1) non-clinical and (2) clinical samples. The second further divided the clinical samples, to assess the level of PSS across the following groups: (1) non-clinical, (2) probable PTSD, (3) mild TBI, (4) amputation injury and (5) any other treatment-seeking samples. This includes individuals referred to a health clinic, but the paper does not specify for which health condition. For example, personnel who accessed treatment through Veterans Affairs (VA). If studies consisted of multiple groups, for example a probable PTSD group and a treatment-seeking group [31], these were categorised into separate subgroups. One study recruited a non-clinical sample, however most of the sample (70.4%) reported probable PTSD therefore this was included within the probable PTSD subgroup [32]. In studies where means and standard deviations (SD) were not reported for the total sample, means for reported subgroups were combined prior to analysis using combined mean formula. A random-effects model was generated due to the high heterogeneity of the included studies. This suggests studies were different in nature, in this case a wide range of samples were included with different clinical presentations. The random-effects approach is deemed a sufficient method for dealing with heterogeneity as it assumes that underlying effects follow a normal distribution. Therefore, the pooled estimate would be the mean or average effect. The effect sizes are assumed to represent a random sample of all possible effect sizes, as compared to fixed-effects, which assumes one true effect size underlies all the studies [33]. Results were displayed using forest plots. Funnel plots were generated to visually assess for publication bias. It is also important to quantify publication bias and this was done using Egger’s test [34].

To examine factors associated with PSS, a narrative synthesis was performed. This approach was chosen due methodological and clinical heterogeneity; for example, variation in sample sizes, target populations and clinical outcomes [35].

## Results

### Study selection

**Fig. 1:**
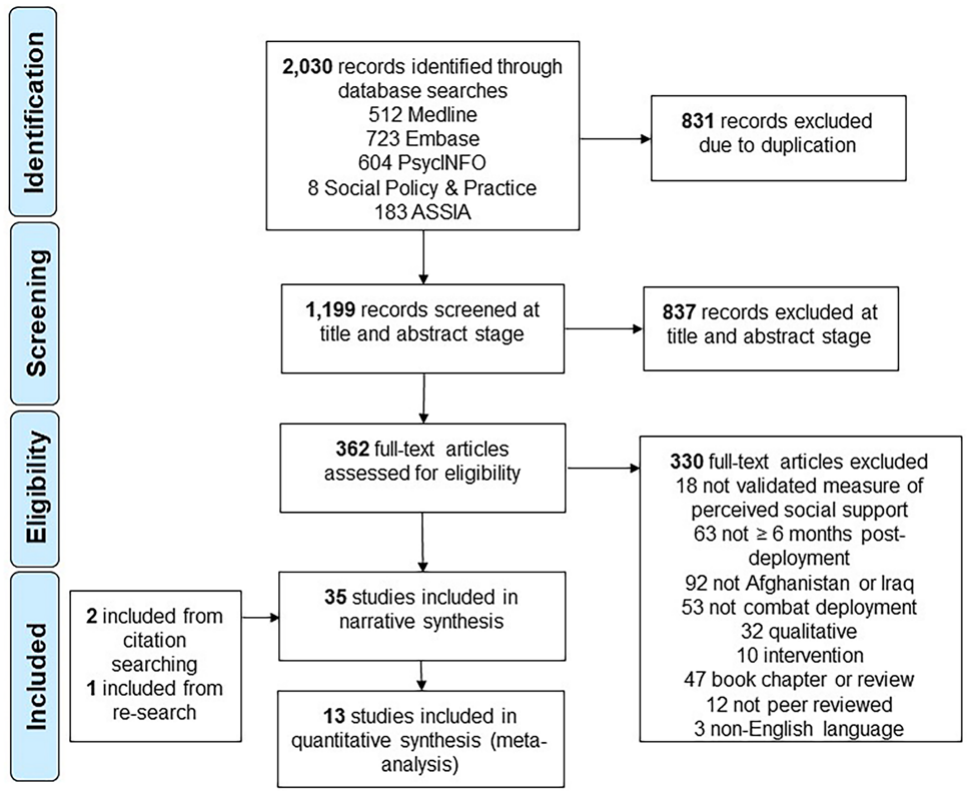
PRISMA flow chart

Figure 1 illustrates our search strategy results. Inter-rater agreement was strong at 96% at both the title and abstract stage, and full-text screening stage.

### Study characteristics

In total, 35 papers published between 2011 and 2023 were included (Table 1). Thirty-one studies were conducted in the US, and the remaining studies were undertaken in the Netherlands [36, 37], Denmark [38] and Norway [39]. Twenty-three studies employed a cross-sectional methodology with the remaining studies being prospective (11 studies) and retrospective cohort studies (1 study). Sample sizes ranged from 45 [40] to 3465 [39] and the mean sample size was 572.5 (SD 706.6). Most samples were predominantly male (31 studies). Studies were made up of non-clinical samples (n = 12) [36–39, 41–49], probable PTSD (n = 8) [29, 32, 50–55], mild TBI (n = 1) [56], physical/amputation injury (n = 3) [17, 40, 57] and all other treatment-seeking samples where health condition is not specified (n = 12) [24, 30, 31, 53–55, 58–63]. One study investigated specific domains of social support (structural, functional), whilst three investigated type of social support (emotional, tangible).

**Table 1:** Study characteristics and quality assessment for included studies

Author, Year	Study design^a^	Sample description	Male, N (%)	Age in years, Mean (SD)^b^	Study information	Quality
Badour et al. 2015 [50]	Cross-sectional	150 US Male Iraq/Afghanistan veterans meeting diagnostic criteria for combat-related PTSD (n = 123, 82%) or subthreshold PTSD (n = 27, 18%)	150 (100.0)	34.9 (9.6)	Participants recruited to 1 of 2 clinical treatment studies after referral to a Southeastern VA medical centre PTSD clinic	Fair
Balderrama-Durbin et al. 2013 [45]	Cross-sectional	76 US active duty Air Force service members who deployed to Iraq, all currently in a committed relationship	70 (92.0)	27.9 (6.1)	Participants recruited to a longitudinal study assessing risk and protective factors across a 1-year deployment to Iraq (from 2009 to 2010). Data collected pre-, during and post-deployment. Current study reports on post-deployment data	Fair
Benetato 2011 [57]	Cross-sectional	56 US Iraq/Afghanistan veterans with major combat-related amputation	53 (94.6)	31.0 (range 22–48). SD not reported	Participants identified through inpatient and outpatient VA records with a 27% response rate. Data collection occurred in 2007, mean 36.4 months post-amputation	Fair
Bernstein et al. 2022 [58]	Cross-sectional	288 US Iraq/Afghanistan veterans and active duty service members, exposed to at least one traumatic event during deployment	257 (89.2)	37.8 (9.2)	Participants enrolled in the Translational Research Center for TBI and Stress Disorders longitudinal cohort study at the VA Boston Healthcare System. Time since deployment ranged from 82.9 to 96.5 months	Fair
Campbell and Riggs 2015 [46]	Cross-sectional	117 US college veterans who deployed to Iraq/Afghanistan, enrolled in college for 5 + months	98 (83.8)	32.5 (7.5)	Participants recruited via email invitations and advertisements. Used data from a larger study examining psychological, social and academic functioning of college student veterans, from 2012 to 2013	Fair
Castillo et al. 2021 [17]	Cross-sectional	429 US Iraq/Afghanistan veterans with major limb trauma	418 (97.5)	18–24 years = 23.6% 25–29 = 34.6% ≥ 30 = 41.8%	Participants recruited from 4 treatment facilities. Data collected between 2003 to 2007. Mean 38.6 months since injury	Fair
Ciarleglio et al. 2018 [59]	Prospective cohort	375 US active duty soldiers, reservists and veterans who deployed to Iraq between 2003 to 2005	356 (95.0)	35.1 (5.9)	Data collected for VA Cooperative Studies Program Study from 2003 to 2014 at 3 timepoints. Post-deployment measures taken 7.5 years following initial Iraq deployment	Fair
Driscoll et al. 2015 [47]	Prospective cohort	460 US Iraq/Afghanistan veterans who endorsed pain for 3 + months	Male: 201 (43.7) Female: 259 (56.3)	Male: 35.5 (10.6) Female: 32.4 (10.7)	Data derived from Women Veterans Cohort Study which included both male and female veterans. Participants recruited via mailings. Data collected between 2008 to 2011	Fair
Eakman et al. 2016 [40]	Cross-sectional	45 US Iraq/Afghanistan veterans with service-related injuries compared to non-veterans (undergraduate students)	Veterans: 23 (88.5) Non-veterans (students): 15 (78.9)	Veterans: 31.6 (8.1) Non-veterans: 27.8 (3.5)	Participants took part in a supported education programme 6 + months prior to completing surveys and had been honourably discharged from the military. Data collected 2013 to 2014	Fair
Eisen et al. 2014 [41]	Prospective cohort	512 US Iraq/Afghanistan veterans	206 (89.9)	Mean (SD) not reported ≤ 25 years: n = 113 (26.5%) 26–34: n = 152 (43.7%) 35–44: n = 162 (23.1%) ≥ 45: n = 85 (6.7%)	Participants recruited 2008 to 2009. 2 timepoints: T1 was 3–12 months post-deployment, T2 was 6–12 months after T1	Good
Fang et al. 2015 [31]	Cross-sectional	1530 US Iraq/Afghanistan veterans	764 (49.0)	37.4 (range 22–69)	Participants were members of the Veterans After-Discharge Longitudinal Registry. Data collection occurred mean 5.3 years post-deployment	Fair
Goetter et al. 2020 [51]	Cross-sectional	223 US veterans or active duty soldiers serving in Iraq/Afghanistan and had combat-related PTSD for at least 3 months with significant impairment (CAPS score ≥ 50)	194 (86.9)	Major Depressive Disorder: 34.3 (8.2) No Major Depressive Disorder: 33.8 (8.5)	Participants recruited between 2011 and 2016 from clinics/PTSD specialty programmes across four sites. Data collected prior to treatment initiation within a randomised controlled trial	Fair
Gros et al. 2019 [52]	Cross-sectional	98 US Iraq/Afghanistan veterans meeting DSM-IV criteria for combat-related PTSD or subclinical PTSD via the CAPS	98 (100.0)	36.0 (8.8)	Participants recruited through referrals from physicians and medical staff at a Southeastern VA medical centre	Fair
James et al. 2013 [42]	Prospective cohort	271 US Iraq/Afghanistan veterans registered for VA healthcare. Less than half had accessed healthcare and those receiving mental health care were excluded, therefore this was a non-clinical sample as stated by the authors	230 (85.0)	31.0 (9.3)	Participants were recruited from VA health care register. T1 occurred 6 months post-deployment, T2 at 12 months and T3 at 24 months	Fair
Kaczkurkin et al. 2016 [53]	Cross-sectional	366 US male Iraq/Afghanistan veterans seeking treatment for PTSD, must have experienced criterion A traumatic event related to combat and met DSM-5 diagnosis	366 (100.0)	Non-Hispanic white: 31.3 (6.7) African American: 37.2 (7.1) Hispanic/Latino: 33.9 (7.1)	Participants were treatment-seeking, through a randomised controlled trial investigating prolonged exposure therapy	Good
Lee et al. 2017 [29]	Prospective cohort	150 US Iraq/Afghanistan veterans with at least subthreshold PTSD symptoms related to combat, and hazardous alcohol use	132 (88.0)	29.5 (7.0)	Participants recruited from VA primary care clinics in New York, referred by primary care staff. Data collected at two timepoints: baseline and 1 year later	Good
Lemaire and Graham 2011 [63]	Cross-sectional	1740 US Iraq/Afghanistan veterans	With Suicidal Ideation: 95 (84.1) Without Suicidal Ideation: 1424 (88.8)	With Suicidal Ideation: 29.4 (8.4) Without Suicidal Ideation: 29.4 (7.5)	Returning veterans were registered with the Houston VA Medical Centre. Data collection occurred between 2004 to 2008, 2–3 years post-deployment	Fair
Lind et al. 2017 [43]	Cross-sectional	133 US Iraq/Afghanistan veterans	116 (88.5)	29.8 (4.7)	Sample included veterans who took part in an ongoing study examining the effects of combat trauma and stress on drinking behaviour. Recruited through the community, VA hospitals and advertising	Fair
Luciano and McDevitt-Murphy 2017 [30]	Cross-sectional	63 US Iraq/Afghanistan veterans	52 (82.5)	36.6 (10.9)	Participants recruited from a VA Medical Centre. Sample representative of VA-using Iraq/Afghanistan veterans	Fair
Matthieu et al. 2017 [49]	Prospective cohort	346 US Iraq/Afghanistan veterans, who served for a minimum of 2 years with honourable discharge and clean criminal record	236 (68.2)	Mean (SD) not reported 22–40 years: n = 286.0 (82.7%) 41–55 years: n = 60.0 (17.3%)	The study evaluated the impact of a civic service programme administered by ‘The Mission Continues’, which involved volunteering for 20 h p/week for 26 weeks at a local non-profit organisation. Data collection occurred from 2011 to 2014	Fair
Meyer et al. 2013 [55]	Cross-sectional	109 US trauma-exposed Iraq/Afghanistan veterans, 55 with PTSD diagnosis on the CAPS	84 (89.0)	37.7 (10.7)	Participants recruited from the Central Texas Veterans Health Care system, through mailings and advertising. Recruitment was targeted toward oversampling those with mental health diagnoses	Fair
Nordstrand et al. 2020 [39]	Cross-sectional	3465 Norwegian military personnel who deployed to Afghanistan between 2001 to 2011	3177 (91.7)	30.0 (9.0)	Military personnel who deployed to Afghanistan between 2001 to 2011 were invited to participate. Response rate was 56.7%. Data collection occurred in 2012, at least 1-year post-deployment	Good
Pollmann et al. 2022 [38]	Prospective cohort	600 Danish army veterans who deployed to Afghanistan, 3 timepoints (pre-deployment, homecoming and 2.5 years post-deployment)	570 (95.0)	Median 23 (IQR 22, 24)	The study combines data from two cohorts of Danish military personnel. Data collection occurred between 2009 and 2013. Measures were completed pre-deployment, after homecoming and 2.5 years post-deployment	Fair
Porcari et al. 2017 [60]	Cross-sectional	325 US Iraq/Afghanistan veterans	280 (86.2)	35.5 (9.7)	Participants were registered for physical or mental health services at the VA. Data collection occurred between 2001 and 2007, at least 1-year post-deployment	Fair
Pugh et al. 2018 [61]	Retrospective cohort	2023 US Iraq/Afghanistan veterans with 3 years in VA care	1093 (54.0)	40.7 (10.3)	A cohort of Iraq/Afghanistan veterans were identified who received VA care between 2007 and 2011. Data collection occurred 2014 to 2015	Good
Scott et al. 2013 [62]	Cross-sectional	634 US Iraq/Afghanistan veterans in VA care	290 (46.0)	37.8 (10.3)	Participants were a sample from a larger study, the Women Veterans Cohort Study, investigating health outcomes in men and women in VA care	Fair
Seidl et al. 2015 [56]	Cross-sectional	95 US Iraq/Afghanistan veterans with combat-related mTBI, defined by the American Congress of Rehabilitation Medicine	92 (97.0)	30.2 (6.5)	Participants were drawn from a sample of 230 veterans who were referred for neuropsychological evaluation at VA sites with possible history of TBI. TBI status confirmed by 2 clinicians	Fair
Smith et al. 2013 [48]	Cross-sectional	96 US Iraq/Afghanistan combat veterans, some were veterans (N = 32, 33.3%) and activity duty, National Guard or reservists (N = 34, 35.4%) Branch of service for the remaining participants was unknown	59 (66.0)	Median 27 (IQR 20, 38)	Recruitment occurred through fliers, undergraduate psychology courses and snowball sampling	Fair
Smith et al. 2017 [44]	Cross-sectional	469 US Iraq/Afghanistan veterans	400 (40.1)	35.3 (10.8)	Participants were obtained from a roster of returning veterans from 2007 to 2009. Response rate 47%	Good
Van der Wal et al. 2020 [36]^c^	Prospective cohort	963 Dutch veterans who served in Afghanistan	878 (91.0)	Mean (SD) not reported < 21 years: n = 130 (14%) ≥ 21 years: n = 831 (87%)	Participants who deployed between 2005 and 2008 were recruited for the Prospective Research in Stress-Related Military Operations study. Assessments completed 1 month before deployment, up to 6 months post-deployment, then 1, 2, 5 and 10 years later	Good
Van der Wal et al. 2022 [37]^c^	Prospective cohort	978 Dutch veterans who served in Afghanistan	893 (91.0)	Mean (SD) not reported < 21 years: n = 136 (14%) ≥ 21 years: n = 840 (86%)	Participants who deployed between 2005 and 2008 were recruited for the Prospective Research in Stress-Related Military Operations study. Assessments completed 1 month before deployment, up to 6 months post-deployment, then 1, 2, 5 and 10 years later	Good
Vasterling et al. 2023 [64]	Prospective cohort	1087 US army soldiers who deployed to Iraq	1011 (93.0)	25.8 (5.9)	Participants who deployed to Iraq at least once between 2003 and 2005 were recruited as part of the Neurocognition Deployment Health Study. Pre-deployment, post-deployment and additional follow-up 1–3 years post-deployment and 5 years + post-deployment	Good
Warrener et al. 2021 [24]	Cross-sectional	86 US male Iraq/Afghanistan veterans	86 (100.0)	36.1 (9.0)	Recruited through flyers at VA medical centre, mean 6 years post-deployment	Fair
Whiting et al. 2016 [32]	Cross-sectional	738 US Iraq/Afghanistan veterans	463 (63.0)	36.0 (8.7)	Participants recruited via internet, media, VA and word-of-mouth sources	Good
Woodward et al. 2018 [54]	Prospective cohort	264 US Iraq/Afghanistan veterans, all who experienced a war-related Criterion A traumatic event for PTSD. 83 with PTSD diagnosis (31.4%)	175 (66.3)	38.8 (9.8)	Participants recruited to a larger study, recruited from Central Texas Veterans Healthcare system using randomised mailings and flyers at VA hospitals. Baseline and T1 (1 year later)	Good

*US* United States, *VA* Veterans affairs, *PTSD* post-traumatic stress disorder, *mTBI* mild traumatic brain injury, *CAPS* Clinician-Administered PTSD Scale, *DSM-IV* diagnostic and statistical manual of mental disorders 4th edition, *IQR* interquartile range

Note: in the US, a veteran is defined as military personnel who have deployed at least once but may still be a serving member of the US Armed Forces

^a^The reported study design is based on how the perceived social support data was used. For example, if the study is a longitudinal cohort, but perceived social support was only measured cross-sectionally, cross-sectional is reported

^b^Mean (SD) is reported, unless otherwise stated

^c^Studies were not independent

### Quality appraisal

Of the 35 included studies, 24 were rated as having “fair” methodological quality and 11 were rated as “good” (Table 1). Lower rated studies tended to have small sample sizes or did not adequately adjust for confounders. Overall, study quality was satisfactory, however the strength of evidence is reduced in cross-sectional studies as the direction of causality cannot be determined.

### Measuring PSS

Seven different instruments were used to examine PSS (see Appendix 2), all of which were validated. The most commonly used measure was the DRRI-PDSS-1 scale [28] (n = 15). Additional measures include the DRRI-PDSS-2 [65] (n = 7), Multidimensional Scale of Perceived Social Support (MSPSS) [66] (n = 4), Interpersonal Support Evaluation List (ISEL) [8] (n = 3), Medical Outcome Study Social Support Survey (MOS-SSS) [67] (n = 4), Oslo Social Support Scale-3 (OSSS-3) [68] (n = 1) and the Provisions of Social Relations Scale [69] (n = 1).

### Level of PSS

**Fig. 2:**
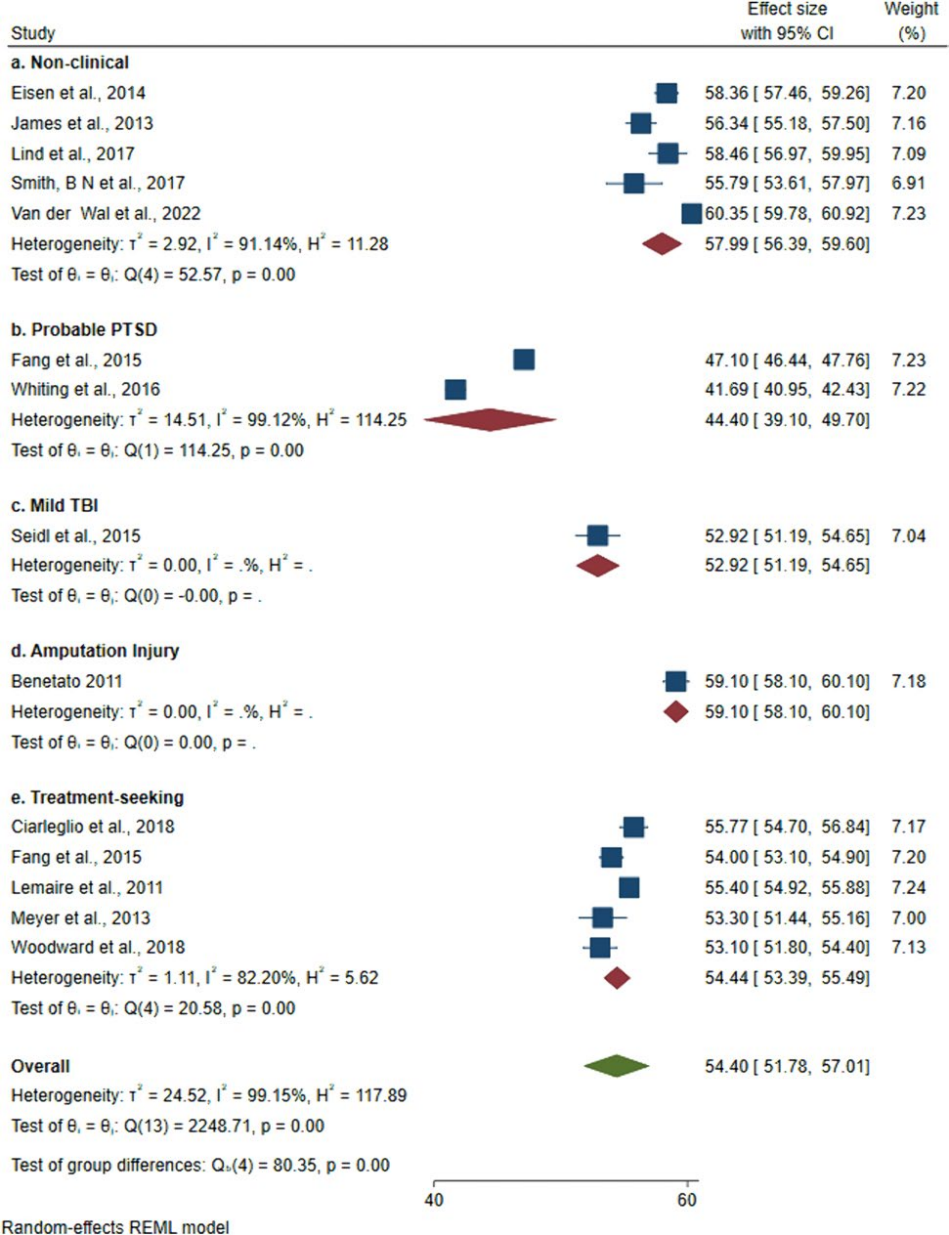
Forest plot showing pooled mean PSS score by sample type. PSS measured using DRRI-PDSS-1 where scores can range from 15 to 75. *PTSD* post-traumatic stress disorder, *TBI* traumatic brain injury

Most studies reported mean level of PSS along with SD whilst four did not. The pooled mean PSS score was 54.40 (95% CI 51.78 to 57.01). Subgroup analyses revealed a higher pooled mean PSS score in non-clinical samples (57.26) compared to clinical samples (52.29) (Appendix Fig. 3). Further subgroup analyses indicated samples with probable PTSD reported the lowest mean level of PSS (44.40), followed by mTBI (52.92), treatment-seeking (54.44) and non-clinical (57.99), whilst an amputation injured sample reported the highest level of PSS (59.10) (Fig. 2). Substantial heterogeneity was observed among included studies (*I*^*2*^ = 99.15%, p < 0.001) meaning a random-effects model was selected. The funnel plots showed no evidence of publication bias (Appendix Fig. 4) as did the findings from Egger’s test (p = 0.68).

### Associated factors

Table 2 summarizes the breadth and strength of evidence for all investigated factors. Findings are presented in Table 3 in more detail. As indicated in Table 3, we identified which studies controlled for confounders. Studies where adjusted effect sizes were reported illustrate that potential confounders have been considered and accounted for. In total, 13 studies adjusted for confounders whilst 22 did not. The confounders varied depending on the outcome of interest. Potential confounders were identified from existing literature and generally included demographic characteristics (age and sex), psychosocial factors (family functioning and unit support), deployment stress and the number of deployments. For the narrative synthesis, factors have been categorised into the following six domains: mental health, social/behavioural, physical health, military, psychological and demographic factors. Next to each header, n denotes the number of papers that examined these factors.

**Table 2:**
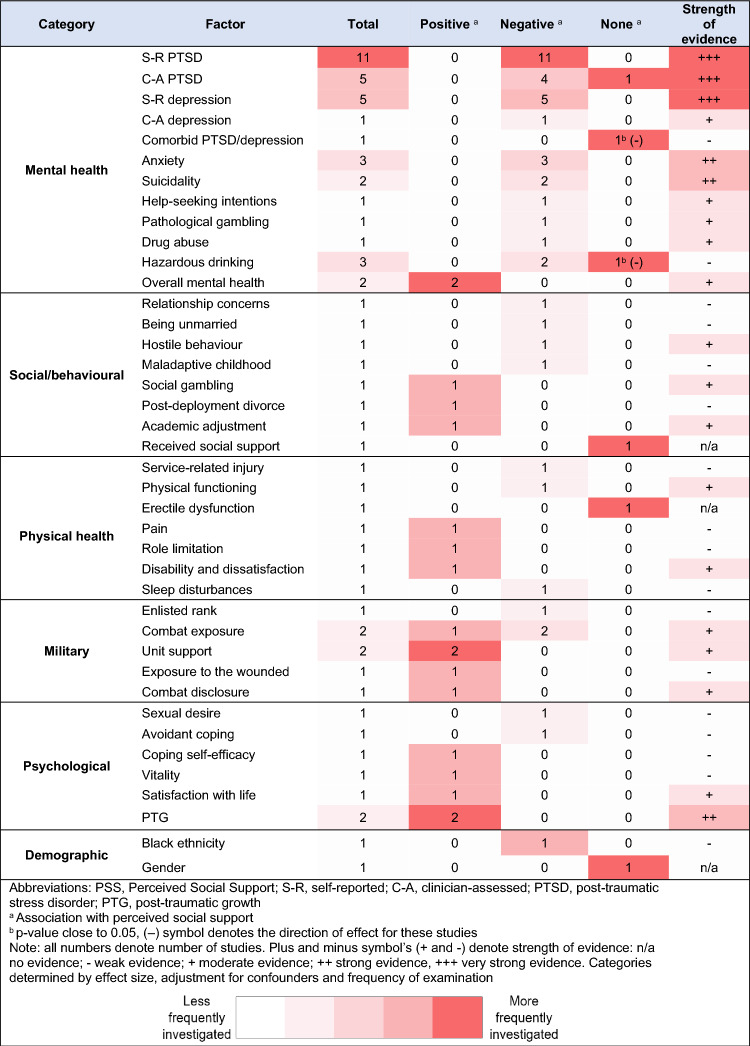
Summary table illustrating breadth and strength of evidence for factors associated/not associated with PSS

**Table 3:** Measure and level of PSS, associated or non-associated factors

Author, Year	PSS measure	Level of PSS, Mean (SD)	Associated or non-associated factor(s)
Badour et al. 2015 [50]	MOS-SSS, range 20–100	56.10 (24.50)	**Associated:** Self-reported sexual desire problems (t = − 4.45, p < 0.001) **Not associated:** Clinician-assessed erectile dysfunction (t = − 1.08, p > 0.05)
Balderrama-Durbin et al. 2013 [45]	MSPSS, range 12–84	Not reported	**Associated:** Self-reported PTSD (β = − 0.36, 95% CI − 2.00 to − 0.48, p < 0.05) Self-reported combat disclosure (β = 0.27, 95% CI 0.04 to 0.67, p < 0.05)
Benetato 2011 [57]	DRRI-PDSS-1, range 15–75	59.10 (7.40)	**Associated:** Self-reported PTG (adjusted β = 0.26, p < 0.01)
Bernstein et al. 2022 [58]	DRRI-PDSS-2, range 10–50	Full sample: 38.71 (8.30) Disability/dissatisfaction: 33.23 (9.29) No disability or dissatisfaction: 41.83 (6.22)	**Associated:** Self-reported disability/dissatisfaction (compared to no disability/dissatisfaction) (AOR 1.07, 95% CI 0.51 to 2.25, p < 0.05)
Campbell and Riggs 2015 [46]	MSPSS, range 12–84	Mean 5.41 (SD 1.08); higher than the theoretical midpoint of 3.5	**Associated:** Self-reported academic adjustment (measures attitudes, motivations, performance and satisfaction towards college) (adjusted β = 0.20, p < 0.05)
Castillo et al. 2021 [17]	MSPSS, range transformed to 0–100	Total 73.70 (11.60) Low (0–70): 27.0% Moderate (71–80): 37.3% High (81–100): 35.7%	None reported
Ciarleglio et al. 2018 [59]	DRRI-PDSS-1, range 15–75	55.80 (10.60)	**Associated:** Clinician-assessed PTSD (AOR 0.92, 95% CI 0.89 to 0.95, p < 0.001) Clinician-assessed depression (AOR 0.92, 95% CI 0.89 to 0.95, p < 0.001) Clinician-assessed anxiety (AOR 0.95, 95% CI 0.93 to 0.98, p < 0.001) **Not associated:** Problem drinking (AUDIT ≥ 8) (OR 0.98, 95% CI 0.95 to 1.00, p = 0.09)
Driscoll et al. 2015 [47]	MOS-SSS, range 20–100	Male Emotional support: 1.97 (1.24) Tangible support: 2.36 (1.44) Female Emotional support: 2.03 (1.36) Tangible support: 2.46 (1.57)	**Associated:** Emotional support negatively associated with combat exposure in men (r = − 0.17, p < 0.05) and women (r = − 0.18, p < 0.05) Tangible support positively associated with combat exposure in women (r = 0.14, p < 0.001) Emotional support positively associated with tangible support for both genders (r > 0.61 for all, p < 0.05) **Not associated:** Gender not associated with emotional (t = 0.52, p = 0.60) or tangible support (t = 0.75, p = 0.46)
Eakman et al. 2016 [40]	DRRI-PDSS-2 with military-related items removed, range 6–30	Veterans with service-related injury: 20.23 (6.31) Non-veterans: 25.08 (4.01)	**Associated:** Veterans with service-related injury reported lower levels of PSS, compared to non-veterans (t = − 2.94, p = 0.01)
Eisen et al. 2014 [41]	DRRI-PDSS-1, range 15–75	For T2 (6–12 months post-deployment): 58.36 (10.34)	**Associated:** Post-deployment PSS at T1 associated with the following factors at T2: Self-reported PTSD (adjusted β = − 0.25, p < 0.001) Self-reported mental health status (higher scores better) (adjusted β = 0.11, p < 0.001) Self-reported alcohol use score (adjusted β = − 0.02, p < 0.05) Self-reported drug abuse score (adjusted β = − 0.01, p < 0.05)
Fang et al. 2015 [31]	DRRI-PDSS-1, range 15–75	Men with PTSD: 48.40 (10.0) Men without PTSD: 54.20 (11.20) Women with PTSD: 45.80 (10.80) Women without PTSD: 53.80 (10.90)	**Associated:** Clinician-assessed PTSD (effect size not reported, p < 0.001)
Goetter et al. 2020 [51]	DRRI-PDSS-1, range 15–75	50.75 (9.18)	**Associated:** Self-reported depressive symptom severity (β = − 0.44, 95% CI − 0.60 to − 0.29, p < 0.001) **Not associated:** Presence vs. absence of comorbid Major Depressive Disorder and PTSD (OR 0.97, 95% CI 0.93 to 1.00, p = 0.066)
Gros et al. 2019 [52]	MOS-SSS, range 20–100	Married: 47.20 (16.00) Post-deployment divorce/separation: 66.10 (27.00)	**Associated:** Post-deployment divorce (r = − 0.41, p < 0.01) Divorced, compared to married (Cohen’s D = 0.85, p < 0.001)
James et al. 2013 [42]	DRRI-PDSS-1, range 15–75	At T2 (24 months post-deployment): 56.34 (9.72)	**Associated:** PSS at T1 associated with self-reported PTSD at T1 (β = − 0.81, p < 0.002) PSS at T2 associated with self-reported PTSD at T2 (β = − 0.39, p < 0.002) and T3 (β = − 0.42, p < 0.002) PSS at T2 associated with self-reported depression at T2 (β = − 0.45, p < 0.002) and self-reported depression at T3 (β = − 0.38, p < 0.002) **Not associated:** PSS at T1 not associated with self-reported depression at T1 (β = − 0.18, p > 0.05) or self-reported alcohol misuse at T1 (β = − 0.01, p < 0.002) PSS at T2 not associated with self-reported alcohol misuse at T2 (β = − 0.22, p < 0.002) or T3 (β = − 0.19, p < 0.002)
Kaczkurkin et al. 2016 [53]	ISEL, range 12–48	Appraisal support for 3 ethnicities range between 10.67–11.41 Belonging range between 11.14–11.94 Tangible support range between 11.68–11.76	**Associated:** Higher PTSD symptom scores associated with lower levels of appraisal (adjusted β = − 0.19, p < 0.05), belonging (adjusted β = − 0.16, p < 0.05) and tangible support (adjusted β = − 0.15, p < 0.05)
Lee et al. 2017 [29]	DRRI-PDSS-1, range 15–75	Not reported	**Associated:** Self-reported PTSD severity at timepoint 1 (β = − 0.35, p < 0.001) and 2 (β = − 0.28, p = 0.008) Maladaptive childhood family environment (β = − 0.21, p < 0.001) Avoidant coping (β = − 0.33, p < 0.001)
Lemaire and Graham 2011 [63]	DRRI-PDSS-1, range 15–75	With suicidal ideation: 46.80 (10.40) Without suicidal ideation: 55.40 (10.30)	**Associated:** Suicidal ideation (OR 0.95, 95% CI 0.92 to 0.98, p < 0.001)
Lind et al. 2017 [43]	DRRI-PDSS-1, range 15–75	58.46 (8.77)	**Associated:** Self-reported sleep disturbances (β = − 0.24, p = 0.021)
Luciano and McDevitt-Murphy 2017 [30]	DRRI-PDSS-1, range 15–75	39.75 (10.53) Note: missing data was substantial	**Associated:** Self-reported unit support (β = 0.34, p < 0.05) Self-reported general health (β = 0.37, p < 0.01) Self-reported pain (β = 0.35, p < 0.01) Self-reported vitality (β = 0.41, p < 0.01) Self-reported role limitation (β = 0.29, p < 0.01) Self-reported physical functioning (β = 0.37, p < 0.01)
Matthieu et al. 2017 [49]	ISEL, range 12–48	T1 (before civic service programme completion): 34.54 (8.37) T2 (after civic service programme completion): 36.54 (7.69)	**Associated:** PSS increased after civic service completion (d = − 0.25, p < 0.001) Self-reported probable depression was associated with PSS after programme completion (adjusted β = − 0.15, p < 0.05) **Not associated:** After programme completion, PSS was not associated with… Self-reported PTSD (β = − 0.09, p > 0.05) Seeking professional treatment for emotional problems (β = − 0.04, p > 0.05)
Meyer et al. 2013 [55]	DRRI-PDSS-1, range 15–75	53.30 (9.90)	**Associated:** Self-reported PTSD negatively associated with PSS (adjusted β = − 0.22, p < 0.0125) **Not associated:** Clinician-assessed PTSD not associated with PSS (adjusted β = 0.17, p > 0.05)
Nordstrand et al. 2020 [39]	OSSS-3, range 5–25	Structural social support: 4.04 (0.88) Functional social support: 18.53 (3.16)	**Associated:** PTG was associated with both functional PSS (adjusted β = 0.23, p < 0.001) and structural PSS (adjusted β = 0.19, p < 0.001)
Pollmann et al. 2022 [38]	MSPSS, range 1–7 (scores summed and divided by number of items)	For 2.5 years post-deployment: Total: 5.73 (0.96) Family: 5.64 (1.20) Friends: 5.58 (1.14) Significant other: 5.98 (1.15)	**Associated:** Negative changes in PSS from pre- to post-deployment were associated with both moderate (AOR 1.99, 95% CI 1.51 to 2.57, p < 0.001) and high levels of PTSD symptoms (AOR 2.71, 95% CI 1.94 to 3.78, p < 0.001) A decline in post-deployment PSS from family (AOR 1.45, 95% CI 1.07 to 2.00, p < 0.05) and friends (AOR 1.40, 95% CI 1.05 to 1.87, p < 0.05) was associated with increased risk of PTSD
Porcari et al. 2017 [60]	ISEL, range 12–48	64.3% seek help for psychological problems from these sources (partner, family, spouse, friends) These were rated as very unhelpful (6.2%), unhelpful (5.5%), neutral (16%), helpful (27.1%), very helpful (9.5%)	**Associated:** Intentions to seek formal help for psychological problems (r = − 0.19, p < 0.01) **Not associated:** Formal help-seeking intentions was not associated with PSS subscales (appraisal, belonging and tangible support)
Pugh et al. 2018 [61]	DRRI-PDSS-2, range 10–50	Mean range from 32.56 to 37.16	**Associated:** Deployment experiences: exposure to wounded people (r = 1.80, p < 0.05) and fear of being killed (r = 2.01, p < 0.05) Being unmarried (r = − 1.14, p < 0.05) Black ethnicity (compared to White) (r = − 1.95, p < 0.05) Enlisted rank (compared to officer) (r = − 1.44, p < 0.05) Mental health problem (r = 4.56, p < 0.05) **Not associated:** Female sex (β = 0.045, p > 0.05), wounded in combat (β = 0.611, p > 0.05)
Scott et al. 2013 [62]	MOS-SSS, range 20–100	Women with alcohol problems (AUDIT ≥ 8): tangible support 65 (28.8); emotional support 72.7 (26.9) Women with no alcohol problems: tangible support 73.3 (27.6); emotional support 82.2 (25.4) Men with alcohol problems (AUDIT ≥ 8): tangible support 65.0 (26.5); emotional support 76.6 (26.4) Men with no alcohol problems: tangible support 73.8 (26.6); emotional support 84.2 (25)	**Associated:** Men and women who engage in hazardous drinking (AUDIT ≥ 8) were more likely to have lower tangible and emotional social support, compared to those with no alcohol problems (Cohen’s d range from −0.69 to −0.29, p < 0.05)
Seidl et al. 2015 [56]	DRRI-PDSS-1, range 15–75	52.92 (8.59)	**Associated:** Satisfaction with life (adjusted β = 0.29, p < 0.05)
Smith et al. 2013 [48]	Provisions of Social Relations scale, range 4–100	77.19 (16.71)	**Associated:** Self-reported post-deployment coping self-efficacy (defined as ability to adapt to demands of societal reintegration following combat) (β = 0.32, p = 0.016) **Not associated:** Received social support (β = 0.29, p > 0.05)
Smith et al. 2017 [44]	DRRI-PDSS-1, range 15–75	Women: 57.42 (9.85) Men: 55.79 (10.92)	**Associated:** Warfare exposure in men (β = − 0.33, p < 0.05) Warfare exposure in women (β = − 0.33, p < 0.05) Deployment PSS in men (β = 0.44, p < 0.05) Deployment PSS in women (β = 0.30, p < 0.05) Relationship concerns during deployment, in men (β = − 0.15, p < 0.05) **Not associated:** Relationship concerns during deployment, in women (β = − 0.12, p > 0.05)
Van der Wal et al. 2020 [36]^a^	DRRI-PDSS-1, range 15–75	60.43 (9.16)	**Associated:** PSS after deployment was associated with a lower increase in PTSD symptoms, meaning those who report higher PSS may experience less change in PTSD symptoms over time (β = − 0.12, p = 0.010) Resilient compared to delayed onset PTSD (AOR 0.95, 95% CI 0.91 to 0.99, p < 0.05)
Van der Wal et al. 2022 [37]^a^	DRRI-PDSS-1, range 15–75	60.35 (9.07)	**Associated:** Lower PSS after returning from deployment was associated with increased risk for… Self-reported agoraphobia 1-year post-deployment (β = − 0.04, p < 0.001) and 10-years post-deployment (β = − 0.05, p < 0.001) Self-reported generalised anxiety at 10-years post-deployment (β = − 0.09, p < 0.001) Self-reported depression at 1-year (β = − 0.23, p < 0.001), 2-years (β = − 0.15, p < 0.001) and 10-years (β = − 0.17, p < 0.001) post-deployment Self-reported hostility at 6-months (β = − 0.07, p < 0.001), 1-year (β = 0.09, p < 0.001), 2-years (β = − 0.04, p < 0.05) and 10-years (β = − 0.05, p < 0.05) post-deployment
Vasterling et al. 2023 [64]	DRRI-PDSS-2, range 10–50	56.70 (9.90)	None reported
Warrener et al. 2021 [24]	DRRI-PDSS-2, range 10–50	Not reported	**Associated:** Self-reported depressive symptoms (β = − 0.48, p < 0.001) Suicidal ideation and behaviour (β = − 0.42, p < 0.001)
Whiting et al. 2016 [32]	DRRI-PDSS-1, range 15–75	Non-gambling: 46.63 (10.26) Social gambling: 45.11 (9.94) Probable pathological gambling: 36.32 (10.63)	**Associated:** Social gambling, compared to non-gambling (AOR 1.02, 95% CI 1.00 to 1.03, p < 0.05) Probable pathological gambling, compared to non-gambling (AOR 0.96, 95% CI 0.93 to 0.99, p < 0.05)
Woodward et al. 2018 [54]	DRRI-PDSS-1, range 15–75	Baseline: 53.10 (10.80) Follow-up 1: 52.30 (11.40)	**Associated:** Baseline PSS predicted self-reported PTSD at 1-year (β = − 0.12, p = 0.04). baseline PTSD predicted PSS at 1-year (β = − 0.15, p = 0.04) Clinician-assessed PTSD at baseline predicted PSS at 1-year (β = − 0.16, p = 0.03) **Not associated:** PSS at baseline did not predict clinician-assessed PTSD at 1-year (β = − 0.05, p = 0.46)

*PSS* perceived social support, *PTSD* post-traumatic stress disorder, *PTG* post-traumatic growth, *AUDIT* alcohol use disorders identification test, *DRRI-PDSS* deployment risk and resilience inventory-post-deployment social support scale, *MSPSS* multidimensional scale of perceived social support, *ISEL* interpersonal support evaluation list, *MOS-SSS* Medical Outcome Study Social Support Survey, *OSSS-3* Oslo Social Support Scale, *CI* confidence interval, *OR* odds ratio, *AOR* adjusted odds ratio

^a^Studies were not independent

#### Mental health (n = 19)

Mental health-related factors were most frequently investigated in relation to PSS. Evidence is strongest for the relationship between PSS and probable PTSD, in terms of breadth and strength. Lower PSS scores were associated with increased self-reported [29, 36, 38, 41, 42, 45, 49, 55] and clinician-assessed PTSD [31, 54, 55, 59], cross-sectionally and at 1, 2, and 10 years post-deployment. Effect sizes ranged from β = −0.81 to −0.25. In one study PSS was not associated with clinician-assessed PTSD [55]. For PSS subscales, lower levels of appraisal, belonging and tangible support were associated with higher PTSD scores [53].

There is strong evidence that lower PSS scores were associated with increased self-reported [24, 37, 42, 49, 51] and clinician-assessed depression [59]. Effect sizes ranged from β = −0.48 to −0.38. However, presence (compared to absence) of comorbid major depressive disorder (MDD) and PTSD was not associated with PSS [51].

Lower PSS scores were cross-sectionally and longitudinally associated with presence of clinician-assessed generalised anxiety disorder [37, 59] and agoraphobia [37]. Additionally, suicidal ideation [63] and behaviour [24], help-seeking intentions for psychological problems [49], probable pathological gambling [32], drug abuse [41] and hazardous drinking [41] were all associated with lower PSS. Hazardous drinking caseness (compared to no hazardous drinking) was associated with distinct types of PSS, specifically, lower tangible and emotional support in both men and women [62]. Conversely, one study found hazardous drinking (defined as AUDIT score ≥ 8) was not associated with PSS [59]. Two studies examined overall mental health status and found higher PSS scores were positively associated with mental health status cross-sectionally [61] and longitudinally [41], where higher scores indicate better mental health.

#### Social/behavioural (n = 10)

Lower PSS scores were associated with increased relationship concerns during deployment [44], being unmarried [61], self-reported hostile behaviour at 1, 2 and 10 years post-deployment [37] and having a maladaptive childhood family environment [29]. Higher PSS scores were associated with social gambling (compared to non-gambling) [32], post-deployment divorce [52], and academic adjustment in student veterans, defined as an ability to handle the demands of college [46]. One study assessed the relationship between two types of PSS and found a positive association between emotional and tangible support in both males and females [47]. Another study found that PSS was not associated with received social support [48].

#### Physical health (n = 4)

Only a few studies investigated the association between lower PSS scores and physical health-related outcomes. Having a service-related injury (compared to not) [40], worsened physical functioning [30] and self-reported sleep disturbances [43] were associated with lower PSS scores, whilst erectile dysfunction was not [50]. In addition, higher PSS scores were associated with increased self-reported pain and role limitation [30], as well as disability and dissatisfaction [58].

#### Military (n = 5)

Military personnel within enlisted ranks (compared to officers) reported lower PSS [61]. Higher combat exposure was associated with lower PSS scores [44]. For subscales, combat exposure was negatively associated with emotional PSS in both men and women and positively associated with tangible PSS in women [47]. Higher PSS scores were associated with increased unit support [30, 44], exposure to wounded people during deployment [61] and combat disclosure, defined as willingness to disclose deployment events [45].

#### Psychological (n = 6)

Lower PSS scores were associated with increased sexual desire problems [50] and avoidant coping styles [29], whilst higher PSS scores were associated with increased coping self-efficacy [48], vitality [30], satisfaction with life [56] and post-traumatic growth (PTG) [57]. Furthermore, one study found PTG was positively associated with both structural and functional PSS [39].

#### Demographics (n = 2)

Only two studies investigated demographic factors in relation to PSS. One found (ex-)military personnel within Black ethnic groups reported lower PSS, compared to White personnel [61]. Another study found no association between emotional or tangible PSS and gender [47].

## Discussion

This study systematically synthesised the evidence base on the levels of PSS, and associated factors, in combat-exposed (ex-)military personnel who were deployed to the Iraq/Afghanistan conflicts. A pooled mean PSS score of 54.40 (95% CI: 51.78 to 57.01) was generated, with probable PTSD samples reporting the lowest levels and an amputation injured sample reporting the highest. A range of mental health, social/behavioural, physical health, military, psychological and demographic factors were associated with different levels of PSS. The associations most frequently examined were low levels of PSS and probable PTSD, depression or anxiety, as well as high levels of PSS and increased PTG or unit support.

### Measuring PSS

Seven different scales were used to examine PSS. Broadly, the scales measure perceptions of the extent to which family, friends, co-workers and the community provide support following deployment. For some scales, types of PSS were examined including emotional, tangible and informational [8, 28, 65, 67, 68]. The DRRI-PDSS-1 was most frequently used and was developed with a sample of Gulf War veterans (1990–1991) [28]. The DRRI-PDSS-2 [65] was created to align with experiences from Iraq/Afghanistan, such as increased risk for insurgency warfare and less emphasis on nuclear exposure [65].

### Self-reported level of PSS

The meta-analysis revealed that the mean DRRI-PDSS-1 score ranged from 41.69 to 60.35, with a pooled mean score of 54.40 (95% CI 51.78 to 57.01). Scores were similar across non-clinical samples but less so for clinical samples. Overall, confidence intervals were small indicating a high degree of certainty. Similar levels of PSS have been reported in US Gulf War veterans (mean 56.69, SD 10.52) [28], where demographic characteristics were similar to those of the studies included in this review. The participants were predominantly white, male and aged between 31 and 40 years. As the DRRI-PDSS is heavily centred around military experience, no general population studies have used this measure therefore comparisons were not possible.

Subgroup analyses revealed a greater level of PSS in non-clinical samples than in clinical samples. Further dividing the clinical samples into subgroups revealed that those with probable PTSD reported the lowest level of PSS (pooled mean 44.50, 95% CI 39.10 to 49.70), whilst an amputation injured subgroup reported the highest level of PSS (59.10, 95% CI 58.10 to 60.10). Several explanations exist for this finding. Amputation injuries are visible and involve intensive rehabilitation, and whilst the PSS questionnaires examine support with daily tasks, participants with a physical injury may access more tangible support due to physical limitations. The rehabilitation process may provide injured personnel with opportunities to build a social network, for example by engaging in collaborative sporting events such as the Invictus games foundation, whilst uninjured personnel may be unaware of these possibilities and have less targeted interventions. This finding may also be explained by PTG, which is positively correlated with PSS and is greater among injured personnel than uninjured personnel [66]. Conversely, PTSD is an invisible injury and may go undiagnosed or undetected. Individuals with PTSD might feel undeserving of support due to stigma, particularly serving personnel who may fear negative consequences for their career [70]. Subgroup analyses for gender, nation and serving status were not possible due to limited data. Samples were predominantly US male personnel and included a combination of serving and ex-serving personnel.

### Associated factors

Probable PTSD and depression were the most robustly assessed factors associated with PSS and were examined cross-sectionally and longitudinally. Comorbid PTSD and MDD was not associated with PSS in one study [46], however the p-value was close to significance at 0.06. One study also found no association between clinician-rated PTSD and PSS although the small sample size may explain why an effect was not detected. The association between PTSD and PSS appears to be bidirectional. One study used a cross-lagged panel approach [54], meaning that reporting lower PSS predicts the later occurrence of PTSD and that having PTSD predicts a reduction in PSS as perceptions are framed by symptomatology. This relationship may occur directly or indirectly. Individuals with PTSD may drive their support networks away through negative thoughts and behaviours, such as avoidance and mistrust [71]. Alternatively, those with low levels of PSS may later experience PTSD due to a lack of support networks with which to discuss traumatic experiences. The disclosure of combat-related experiences is associated with reduced PTSD [45]. There is also moderate evidence for the association between anxiety and lower levels of PSS, which may be explained by similar mechanisms such as social withdrawal.

Turning attention to physical health-related factors, higher PSS scores were associated with increased pain and role limitations [30], as well as disability and dissatisfaction [58]. Individuals who report high levels of pain, role limitations and disability may, in turn, require more tangible support and assistance with daily tasks. It appears that individuals affected by these physical difficulties can still access support despite these challenges. This highlights the importance of measuring subdomains of PSS, for example, treating tangible and emotional support as distinct entities. These findings contradict those of a non-military sample with rheumatoid arthritis in which lower levels of PSS were linked to greater pain intensity [72]. However, the study included within this review had substantial missing data. This could also be condition specific, as rheumatoid arthritis is chronic and debilitating therefore certain symptom profiles may impact PSS differently. This review also revealed that lower PSS scores were associated with more sleep disturbances [43]. This may be because PSS protects against social isolation and increases feelings of connectedness leading to positive health behaviours. Sleep disturbances are also concomitant with other physical and mental health problems [73].

Alongside mental and physical health-related factors, cross-sectional evidence suggests that PSS is associated with a range of social/behavioural factors. Lower PSS was associated with being unmarried [61], whilst higher PSS was associated with greater post-deployment divorce [52]. Those who have recently experienced a divorce may report greater levels of PSS as support networks are more prevalent during times of stress [74]. Individuals might lean on other sources of support such as friends and family in their partners absence.

This review also found evidence to suggest PSS is associated with some psychological, military and demographic factors. Within these topical domains, unit support and PTG have been the most investigated and are positively associated with PSS [30, 57]. This finding aligns with existing research in the general population, as positive associations have been found between PTG and social support [75].

The findings were mostly in line with other populations. In general, and in clinical populations, PTSD [76], depression [13, 77], anxiety [13], suicidal ideation [78] and poor sleep quality [77, 79] are all negatively associated with PSS. Conversely, gender differences in PSS are established within the general population [80], but only one study in this review examined this topic and found no association [81]. Despite this, the p-value was close to 0.05, albeit not significant, which may be indicative of a type II error defined as failure to reject the null hypothesis.

Overall, higher levels of PSS were related to better health and wellbeing outcomes after deployment. However, PSS may not always be beneficial as one study found that high levels of PSS were associated with reduced professional help-seeking for psychological problems. Individuals may feel sufficiently supported by their social network and refrain from seeking professional support when it may benefit them. One study showed that 86% of a sample of United Kingdom (UK) (ex-)military personnel sought informal sources of support for mental health-related problems, whilst only 55% accessed medical support [82].

### Limitations of the evidence base

Most studies were rated as having fair methodological quality; whilst this was deemed sufficient for inclusion within this review, most had a cross-sectional design and longitudinal research was lacking. Longitudinal research would provide an opportunity to examine variables across multiple time points and to determine the direction of the relationship between variables; for example, PSS causing a change in mental health. Therefore, the causal direction of the relationship between PSS and health-related factors cannot be determined in most cases. Second, less than half of the included studies adjusted for confounders showing a potential source of bias. When running unadjusted analyses, it is not clear to what extent other variables may play a role in the relationship between the exposure and the outcome. This is especially important in social epidemiology, where there are numerous variables that are potentially intertwined, and significant collinearity between these variables. Third, few studies have separated PSS into distinct domains, such as source (family and friends) or type of support (emotional and tangible). This is important for gaining an understanding of the differential contributions of subdomains of PSS, as evidence suggests that they may have differing consequences [83]. Fourth, few studies in this review used the updated DRRI-PDSS-2 measure, which was specifically designed for those who deployed to Iraq/Afghanistan; therefore, the meta-analysis was centred around the DRRI-PDSS-1. This should be kept in mind when comparing the findings across studies using different measures and over different time periods. Despite this, only 20% of the questionnaire items from the DRRI-PDSS V1 were revised to create V2, therefore much of it remains the same as before. Fifth, no studies have investigated associations between PSS and injury severity or PTSD symptom profiles. Finally, the data are predominantly derived from US samples (n = 31). The experience of PSS may differ between nations due to differences in social, political and military experiences. Therefore, our reported findings may reflect the specificities of the US military and may not be generalisable to different cultures or contexts.

### Strengths and limitations of the review

The review included studies in which a validated measure of PSS was used. A comprehensive list of search terms were used, and we included concepts related to PSS such as comradery. This review focused on functional measures of PSS, that indicated the quality of PSS, as opposed to structural measures, such as number of social contacts. Functional support is recommended within the social support literature as a more useful source for examining support and it is a better predictor of wellbeing outcomes than structural measures [11].

This review is also subject to various limitations. The review did not examine structural dimensions of social support. Although functional PSS measures are beneficial for understanding the quality of support, they may be influenced by individual differences in perception, memory and judgement [12]. What one person considers to be supportive behaviour may differ from another. Stressful events, such as combat deployment, may challenge one’s belief system and decrease their perception of support, despite it being present. This has been illustrated by the deterioration-deterrence model [74]. This model proposes that the impact of traumatic events on mental health occurs both through social network disruption and a decrease in one’s perception of support. The buffering impact may also be impaired by the person’s ability to process support they receive and relate meaningfully to another individual.

In addition, the review did not include grey literature therefore increasing the risk of publication bias. However, to reduce this concern, we generated funnel plots and conducted Egger’s test which showed no evidence of publication bias. We also included non-statistically significant findings from the included studies where p > 0.05. Finally, the review focused only on quantitative, observational data. Qualitative studies may provide more subjective insights into how individuals perceive their social support. As described previously, functional support is contingent upon perception and subjectivity. Therefore, determining how (ex-)military personnel experience their support is important for further enquiry.

### Implications

Understanding whether these associations are reflected in UK samples will be an important addition to the field. Future research should investigate PSS in (ex-)military personnel from other countries, as the evidence is heavily weighted toward US samples. The experience of PSS may differ in UK samples due to different deployment experiences and sociocultural trends, such as greater alcohol consumption among UK personnel [84]. Studies should also distinguish between sources of support and examine associations with physical health-related outcomes, such as injury severity, as well as military factors.

Based on the review findings, there is sufficient evidence to make recommendations regarding mental health-related factors for the US population. The factors within other categories were based on single and mostly cross-sectional studies. Yet, the strength and breadth of evidence is robust for the relationship between post-deployment PSS and both PTSD and depression. Policies should highlight the importance of PSS, not just immediately after transition from military to civilian life, but earlier on in service to reduce the negative impact after leaving, and for 10 +  years into the future. In addition, policies should be targeted towards enhancing people’s feelings of being supported during challenging times. Psychosocial interventions should include avenues to access social support and enhance understanding of how to foster high quality relationships. Furthermore, developing more peer-to-peer support programmes and highlighting opportunities for societal integration would be beneficial to the individual and society. Increasing levels of PSS may reduce the strain on military mental health services. The UK Government’s Office for Veterans’ Affairs (OVA) strategy for veteran’s has highlighted the importance of communities and relationships for reducing social isolation. The findings from this review hope to pave the way for more UK research in this area to establish policy and practice recommendations.

## Conclusions

This review aimed to synthesise research on the level of PSS, and associated factors, in combat-exposed (ex-)military personnel who deployed to Iraq/Afghanistan. Level of PSS was lowest in samples with probable PTSD and highest in an amputation injured sample. PSS was negatively associated with mental health-related factors, including PTSD, depression and anxiety, and positively associated with psychosocial factors, including PTG and unit support. Findings from this review highlight the importance of maintaining adequate levels of PSS following deployment. The findings suggest that (ex-)military personnel who deployed on a combat operation to Iraq/Afghanistan may benefit from interventions to improve PSS, to drive positive health outcomes and protect against negative outcomes.

## Supplementary Information

Below is the link to the electronic supplementary material.

Supplementary file1 (DOCX 186 KB)

## Footnotes

1 The term (ex-)military describes both those currently serving in the military and those who have left and returned to civilian life.
