# Supplementary material for: Level of perceived social support, and associated factors, in combat-exposed (ex-)military personnel: a systematic review and meta-analysis
Source: Soc Psychiatry Psychiatr Epidemiol. 2024 May 21;59(12):2119–43. doi: 10.1007/s00127-024-02685-3 (PMC11522048; doi:10.1007/s00127-024-02685-3)

**Level of perceived social support, and associated factors, in combat-exposed (ex-)military personnel: a systematic review and meta-analysis**

Laura E. Grover^1*^, Charlotte Williamson^1^, Howard Burdett^1^, Laura Palmer^1^ and Nicola T. Fear^1,2^

^1^King’s Centre for Military Health Research, King’s College London, London, SE5 9RJ, UK; ^2^Academic Department of Military Mental Health, King’s College London, London, SE5 9RJ, UK

***Corresponding author email:** [laura.grover@kcl.ac.uk](mailto:laura.grover@kcl.ac.uk)

**Appendices**

**Appendix 1 - Search Terms**

| **Concept** | **Search terms** |
| --- | --- |
| **Concept A:** Military personnel.ti,ab | **OR** veteran*.ti,ab **OR** armed services.ti,ab **OR** armed forces.ti,ab **OR** army.ti,ab **OR** navy.ti,ab **OR** marines.ti,ab **OR** infantry.ti,ab **OR** ex-servi*.ti,ab **OR** ex-forces.ti,ab **OR** ex-military.ti,ab **OR** combatant*.ti,ab **OR** soldier*.ti,ab **OR** air force.ti,ab **OR** servicemen.ti,ab **OR** servicewomen.ti,ab **OR** paratrooper.ti,ab |
| **AND** |  |
| **Concept B:** Combat*.ti,ab | **OR** warfare.ti,ab **OR** battlefield.ti,ab **OR** deploy*.ti,ab **OR** conflict.ti,ab **OR** active service.ti,ab **OR** Afghanistan.ti,ab **OR** Iraq.ti,ab **OR** injur*.ti,ab **OR** amputation.ti,ab **OR** amputees.ti,ab **OR** trauma.ti,ab **OR** blast injur*.ti,ab **OR** traumatic injur*.ti,ab **OR** wounded.ti,ab **OR** wounding.ti,ab **OR** battle-injured.ti,ab **OR** war-related injuries.ti,ab |
| **AND** |  |
| **Concept C:** Perceived Social Support.ti,ab | **OR** perception adj3 support.ti,ab **OR** emotional support.ti,ab **OR** compassionate support.ti,ab **OR** social connectedness.ti,ab **OR** social integration.ti,ab **OR** social isolation.ti,ab **OR** functional support.ti,ab **OR** network adj3 support.ti,ab **OR** subjective support.ti,ab **OR** quality adj3 support.ti,ab **OR** personal support.ti,ab **OR** confiding relationship.ti,ab **OR** comrade*.ti,ab **OR** camaraderie.ti,ab **OR** friends*.ti,ab **OR** community support.ti,ab **OR** social activit*.ti,ab **OR** social satisfaction.ti,ab **OR** social participation.ti,ab **OR** social network*.ti,ab **OR** social cohesion.ti,ab **OR** social contacts.ti,ab **OR** peer support.ti,ab **OR** unit support.ti,ab **OR** unit cohesion.ti,ab |

**Appendix 2 - Measures of PSS**

| **Name of PSS measure** | **Number of studies** | **Description of measure** |
| --- | --- | --- |
| DRRI-PDSS-1 [28]  DRRI-PDSS-2 [63] | 1: 15 (13 included in meta-analysis)  2: 7 | 1: 15-item questionnaire with 5-point Likert scale.  2: 10-item questionnaire with 5-point Likert scale.  Measures the extent to which family, friends, co-workers, employers and the community provide emotional and instrumental assistance. Questions include: “the reception I received when I returned from deployment made me feel appreciated”; “I am carefully listened to and understood by family or friends”. 20% of the items from V1 were revised to create V2. |
| MSPSS [64] | 4 | 12-item questionnaire with 5-point Likert scale, divided into 3 groups relating to source of support: family, friends and significant other. Each group consists of 4 items. Questions include: “there is a special person who is around when I am in need”; “my friends really try to help me”. |
| ISEL [8] | 3 | 40-item questionnaire with 4-point Likert scale, divided into the following subscales: tangible, belonging, self-esteem and appraisal support. Questions include: “there are several people that I trust to help solve my problems”; “when I feel lonely, there are several people I can talk to”. |
| MOS-SSS [65] | 4 | 20-item questionnaire with 5-point Likert scale, measuring 5 dimensions of social support: emotional, informational, tangible, positive social interaction and affectionate support. Questions based on perceived availability of functional social support, for example “how often is the following kind of support available: someone to give you advice about a crisis; someone who hugs you; someone to make you feel loved”. |
| OSSS-3 [66] | 1 | 3-item questionnaire with 5-point Likert scale. Questions include: “how many people are so close to you that you can count on them if you have great personal problems?”; “how much interest and concern do people show in what you do?”; “how easy is it to get practical help from neighbours if you should need it?” |
| Provisions of Social Relations Scale [67] | 1 | 25-item questionnaire with 4-point Likert scale. Measures the extent to which one is loved, esteemed, and part of a network in which others can be counted on when necessary. |
| Abbreviations: PSS, Perceived Social Support; DRRI-PDSS, Deployment Risk and Resilience Inventory-Post-deployment Social Support scale; MSPSS, Multidimensional Scale of Perceived Social Support; ISEL, Interpersonal Support Evaluation List; MOS-SSS, Medical Outcome Study Social Support Survey; OSSS-3, Oslo Social Support Scale | | |

**Appendix Fig 3 - Level of PSS, Clinical and Non-Clinical Subgroup Analysis**

Forest plot showing pooled mean PSS scores for clinical and non-clinical samples. PSS measured using DRRI-PDSS-1 where scores can range from 15-75.

**
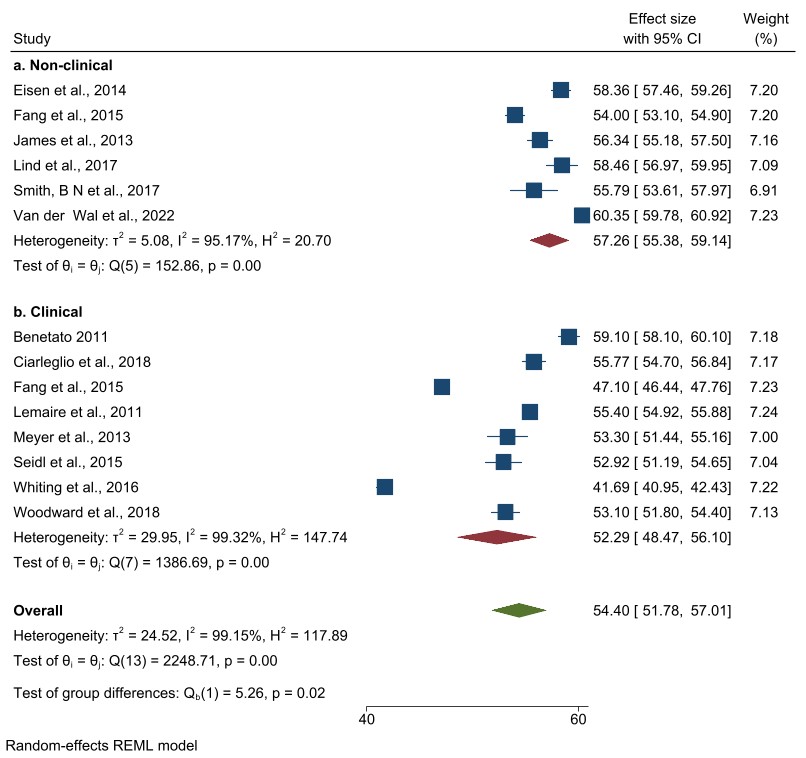
**

**Appendix Fig 4 - Funnel Plot for Meta-analysis**


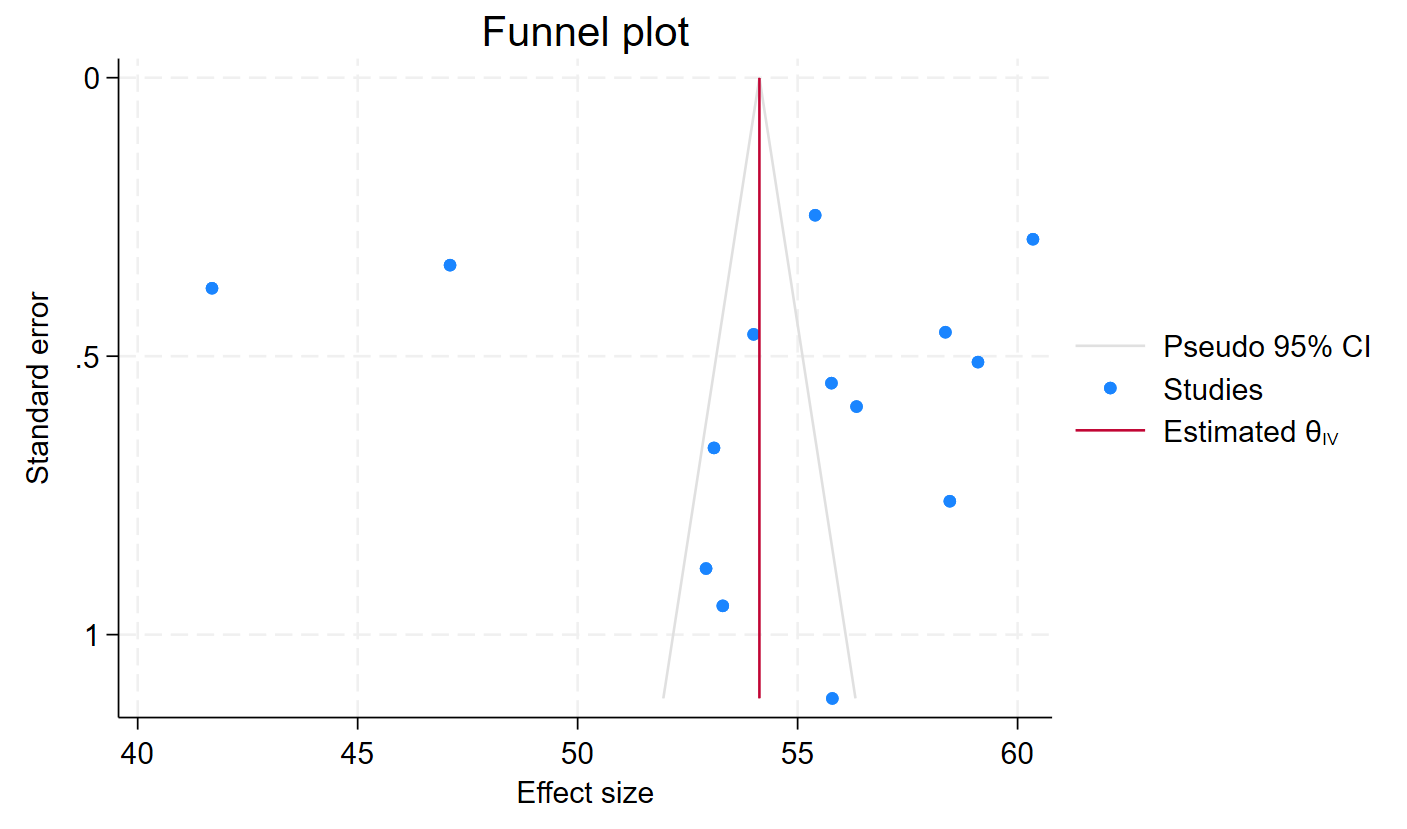

Supplement: Supplementary file 1 — Supplementary file1 (DOCX 186 KB) [file 127_2024_2685_MOESM1_ESM.docx]
